# High-concentrate feeding upregulates the expression of inflammation-related genes in the ruminal epithelium of dairy cattle

**DOI:** 10.1186/s40104-016-0100-1

**Published:** 2016-07-29

**Authors:** Ruiyang Zhang, Weiyun Zhu, Shengyong Mao

**Affiliations:** Laboratory of Gastrointestinal Microbiology, College of Animal Science and Technology, Nanjing Agricultural University, Nanjing, 210095 Peoples Republic of China

**Keywords:** Dairy cows, Gene expression, Microarray, Subacute ruminal acidosis

## Abstract

**Background:**

The objective of this study was to characterize the mRNA expression profile related to rumen epithelial inflammation through the in vivo and in vitro experiments. In the in vivo experiment, rumen papillae were collected from four dairy cows adapted to either a 40 % (LC) or 70 % (HC) concentrate feeds for microarray analysis.

**Results:**

Results showed that 245 differentially expressed genes (DEGs) were detected in the cows fed the HC relative to the LC diet. The DEGs were first annotated, and results revealed that the expression of inflammation-related genes, including *IL-1β*, *IL-2*, *IL-22*, *CCL19*, *CCL8*, *CX3CR1*, *CXCL6*, *INHBE*, *LEPR*, *PRL*, and *TNFRSF9* found in the cytokine-cytokine receptor pathway were up-regulated in the HC-fed cows, indicating local inflammation in the rumen epithelium was triggered. The expression of *IL-1β*, *IL-2*, and *IL-6* was further validated by qRT-PCR. To demonstrate whether there were relationships between cytokine mRNA expression and ruminal factors (pH and LPS), the isolated ruminal epithelial cells were cultured in vitro. Results showed that the mRNA expression of *IL-1β*, *IL-2*, *IL-6*, and *IL-8* increased after the LPS treatment, while low-pH treatment elevated the mRNA expression of *TNF-α*, suggesting that low-pH coupled with higher levels of LPS in rumen of cows fed the HC may be mainly responsible for the triggered local ruminal inflammation.

**Conclusions:**

Our results indicate that ruminal local inflammation response might be triggered during HC feeding, and these findings also enhance the knowledge of rumen epithelial adaptation to HC at the molecular level.

**Electronic supplementary material:**

The online version of this article (doi:10.1186/s40104-016-0100-1) contains supplementary material, which is available to authorized users.

## Background

Feeding ruminants diets rich in fermentable carbohydrates alters ruminal microbiota composition and metabolism, resulting in the accumulation of volatile fatty acids (VFAs) and leading to a dramatic depression of the rumen pH [1, 2]. Long-term high-concentrate feeding likely causes the rumen metabolic disorder commonly termed subacute ruminal acidosis (SARA), which is defined as the daily duration time of ruminal pH remains below 5.6 or 5.8 [3, 4]. SARA has been shown to depress ruminal diet digestibility, feed intake, milk production, and milk fat and has become a significant economic issue in the dairy industry [3, 5].

Dairy cattle health can be compromised by grain-induced SARA since it has been associated with rumenitis, metabolic acidosis, lameness and hepatic abscesses [3, 5]. Many previous reports revealed that grain-induced SARA increased the levels of acute phase proteins such as haptoglobin (Hp) and serum amyloid A (SAA), which indicate a systemic inflammatory response in peripheral blood [6, 7]. This systemic inflammation is considered to be related with the diet-induced massive disruption of rumen epithelial barrier function [8] and increased permeability of the rumen epithelium [9], which aids microbes and immunogenic compounds to transmigrate into portal circulation [7], and further induces systemic inflammation. The principle mechanisms responsible for disrupting the barrier function of the rumen epithelium during SARA are not clear. A study that used isolated rumen and colon tissue from steers demonstrated that LPS and decreased pH may act synergistically to disrupt the epithelial barrier function and then breach the epithelial barrier [10]. Once the epithelium has been breached, the cells of mucosa-associated lymphoid tissue, which are found throughout the digestive mucosa and consist of clusters of white blood cells including innate lymphoid cells and mast cells [11, 12], respond by triggering local inflammation of rumen epithelium and altering cytokine production [11, 13]. Currently the structural transformation of the rumen epithelium during grain-induced SARA has been characterized [8, 14], but inflammation of rumen and the molecular mechanisms that trigger these events are not very clear.

In this study, we hypothesized that a local inflammatory response in the rumen epithelium occurred when cows were fed a high-concentrate diet, and therefore, mRNA expression of genes involved in inflammation would be up-regulated. The first objective of the this study was to characterize the mRNA expression of genes involved in inflammation in the rumen papillae from dairy cows fed high-concentrate diets using bovine genome microarray. The second objective of this study was to explore the relationship between expression of genes related to cytokines and ruminal factors.

## Methods

### Animals, experimental design, and diets

The experimental procedures and animal care conditions were approved by the Experimental Animal Welfare Ethics Committee of Nanjing Agricultural University.

This study was part of a larger experiment investigating the impact of subacute ruminal acidosis adaptation on rumen health in dairy cattle. A detailed description of the experimental design has been previously described [15]. Briefly, four ruminally cannulated Holstein cattle (average body weight, 460 ± 16.4 kg; 84 ± 25 d in milk at the beginning of the trial) were used in a 2 × 2 crossover design trial with 2 cows in each square and 2 periods. All animals were fed a low-concentrate diet (30 % concentrate feed, DM basis) for 3 weeks before the start of this experiment. The dietary treatments were a low-concentrate diet (LC; 40 % concentrate feed, DM basis) and a high-concentrate diet (HC; 70 % concentrate feed, DM basis) (Additional file 1: Table S1). Each experimental period was 21 d, with the first 11 d used for diet adaptation and 10 d of measurements. For the HC group, the dietary concentrate level was stepped up (by approximately 15 percentage units/d compared with the LC diet) during the first 2 d. Throughout the experimental period, the cows received a total mixed ration (TMR), *ad libitum* to about 5 % orts. Cows were housed in tie stalls and had free access to water.

### Blood and rumen papillae samples

Blood samples were taken from the coccygeal vein at 0 and 4 h following the morning feeding on d 12, 17, and 21 of each experimental period. Blood hematological analysis was carried out using an automated haematological analyser (Sysmex K-1000D; Sysmex, Tokyo, Japan), and blood chemistry was measured by Vitros 250 Chemistry System (Ortho Clinical Diagnostics, Markham, Canada).

Rumen papillae samples were collected at 4 h after the morning feeding on d 21 of each experimental period. The ventral sac of the rumen was chosen for collected site [16]. The rumen papillae were excised (approximately 150 mg) from the rumen, washed 20 times in ice-cold PBS, and then frozen immediately in liquid nitrogen until the RNA was isolated.

### Total RNA preparation

Total RNA was isolated from about 100 mg of frozen rumen tissues using TRIzol (Takara Bio, Otsu, Japan) following the manufacturer’s instructions. Qualified total RNA was further purified with the RNeasy mini kit (Qiagen, Hilden, Germany) and RNase-Free DNase Set (Qiagen, Hilden, Germany). The RNA concentration was determined using a NanoDrop spectrophotometer ND-1000UV-Vis (Thermo Fisher Scientific, Madison, WI). The RNA quality was routinely checked by measuring the optical density (260/280 and 260/230 ration). The total RNA from the rumen papillae was amplified and labelled with the Low Input Quick Amp Labeling Kit, One-Color (Agilent technologies, Santa Clara, CA), and the labelled cRNA was purified with RNeasy mini kit (Qiagen, Hilden, Germany).

### Microarray hybridizations and data analysis

The hybridization of the cRNA was performed with Agilent Whole Bovine Genome Oligo (4 × 44 K) Microarrays using the Gene Expression Hybridization Kit (Agilent Technologies) according to the manufacturer’s protocols. After washing in staining dishes, hybridized slides were scanned with the Agilent Microarray Scanner (Agilent Technologies) with the default setting. Raw data were normalized by Quantile algorithm, Gene Spring Software 11.0 (Agilent technologies).

### Isolation and culturing of ruminal epithelial cells

Ruminal epithelial cells were isolated using the modified serial tryptic digestion procedure as described previously [17]. Rumen papillae were taken from the ventral blind sac of healthy lactating Holstein cows (*n* = 3). The cows were fed grass hay. During isolation, each fraction was examined under a phase contrast microscope. The fractions that contained increasing numbers of cells from the stratum basal and the stratum spinosum of the rumen epithelium were pooled. The collected cells were seeded into 25-mL cell culture flasks and cultured with DMEM (Corning Cellgro, Manassas, VA) supplemented with 10 % fetal bovine serum (Corning Cellgro, Manassas, VA) and 1 % antibiotic/antimycotic solution at 38 °C under 5 % CO_2_ for 24 h. Thereafter, the cells were allocated for the following treatments: (1) pH 7.4; (2) pH 5.5; (3) pH 7.4 + 10 μg/mL LPS; (4) pH 5.5 + 10 μg/mL LPS. After incubation for 24 h, the cells were collected and stored at - 80 °C for further analysis. All reagents used were purchased from Sigma (St. Louis, MO) unless indicated otherwise.

### Quantitative RT- PCR and data analysis

Microarray results were validated by quantitative RT-PCR, and the relative expression of cytokines in an in vitro experiment was also conducted by qRT-PCR. Steele et al. [16] reported that *GAPDH* always displayed a minor variation in rumen epithelium samples of dairy cows. Thus, in the present study, the gene *GAPDH* was selected and used as a housekeeping gene. The sequences, amplicon sizes, Gene ID and references of primers used in the present study are listed in Table 1. The dissociation curves obtained from PCR amplification of each target genes (including *GAPDH*) exhibited a unique peak to verify the presence of a single product. Extracted RNA (1 μg) previously used for the microarray analyses was converted into cDNA using a ®PrimeScript RT Reagent Kit with gDNA Eraser (Takara Bio, Otsu, Japan). PCR analyses were performed in triplicate in a final volume of 20 μL reactions containing 10 μL ®SYBR Premix Ex Taq (Takara Bio, Otsu, Japan), 2 μL RT product, 0.4 μmol/L of each forward and reverse primers for the target genes, 0.4 μL ROX Reference Dye (Takara Bio, Otsu, Japan) and 6.8 μL dH_2_O. Reactions were run in a StepOne Plus Realtime PCR System (Applied Biosystems). The thermal cycling parameters were as follows: 30 s at 95 °C, 40 cycles of denaturation at 95 °C for 5 s followed by 30 s annealing at 60 °C. Gene expression of cytokines was normalized to housekeeping gene *GAPDH* (ΔCt = Ct_target_ - Ct_GAPDH_), and the relative expressions, compared with the control group, were calculated using the 2^-ΔΔCt^ method.

**Table 1:** Primers for real-time quantitative PCR

Gene Name	Gene ID	Primer sequence (5′ → 3′)	Amplicon Size,bp	Reference
*IL-1β*	NM_174093.1	For: AACCGAGAAGTGGTGTTCTGC	167	[18]
		R: TTGGGGTAGACTTTGGGGTCT		
*IL-2*	NM_180997.2	For: ACATTTGACTTTTACGCGCCCAAG	307	[19]
		R: AATGAGAGGCACTTAGTGATC		
*IL-6*	NM_000600.3	For: GGAGGAAAAGGACGGATGCT	227	[18]
		R: GGTCAGTGTTTGTGGCTGGA		
*IL-8*	NM_173925	For: CCTCTTGTTCAATATGACTTCCA	170	[18]
		R: GGCCCACTCTCAATAACTCTC		
*IL-12β*	U11815	For: AGGTCGTGGTAGAAGCTGTG	276	[20]
		R: CCTTGTGGCATGTGACTTTG		
*TNF-α*	NM_173966.2	For: CTTCTGCCTGCTGCACTTCG	156	[18]
		R: GAGTTGATGTCGGCTACAACG		
*GAPDH*	NM001034034	For: GGGTCATCATCTCTGCACCT	176	[21]
		R: GGTCATAAGTCCCTCCACGA		

### Statistical analyses

Data for blood chemistry and hematology parameters were analysed by using the general linear model (GLM) procedure in SPSS Version 18 (SPSS, Chicago, IL, USA), according to the model shown below: y_ijkmn_ = μ + S_i_ + C(S)_ij_ + P_k_ + T_m_ + (ST)_im_ + e_ijkmn_, where μ is the overall mean, S_i_ is the diet treatment (*i* = 1–2), C(S)_ij_ is the random effect of cow j (*j* = 1–4) nested within the diet i, P_k_ is the fixed effect of period k (*k* = 1–2), T_m_ is the fixed effect of day m (*m* = 1–3), (ST)_im_ is the fixed effect of the diet by day interaction, and e_ijkmn_ is the random residual error. Significance was declared at *P* < 0.05.

Statistical calculations for the microarray data were conducted by GLM procedure in SPSS Version 18 (SPSS, Chicago, IL, USA), according to the model shown below: y_ijk_ = μ + D_i_ + P_j_ + (DP)_ij_ + e_ijk_, where μ is the overall mean, D_i_ is the diet treatment (*i* = 1–2), P_j_ is the fixed effect of period j (*j* = 1–2), (DP)_ij_ is the interaction between treatment and experimental period, and e_ijk_ is the random residual error. Significance was declared at *P* < 0.05, and a tendency was considered to exist at 0.05 ≤ *P* < 0.10. Differentially expressed genes (DEGs) were defined by genes whose fold-change in expression (the LC group vs. the HC group) was equal to or higher than 1.5-fold and *P* < 0.05. DEGs and heat-map analysis were performed by SAS software from SHANGHAI BIOTECHNOLOGY CORPORATION eBioService (http://www.ebioservice.com). For better understanding of DEGs, overrepresentation analysis of Gene Ontology (GO) terms and pathway in the Kyoto Encyclopedia of Genes and Genomes (KEGG) database was performed. Correlation analysis was performed using GraphPad Prism 5 software (Graphpad Software, San Diego, CA).

Data obtained from the in vitro cell culture experiment were analyzed by GLM procedure of SPSS Version 18 (SPSS, Chicago, IL, USA) to determine the effects of LPS and pH on the mRNA expression of cytokines, according to the model shown below: y_ijk_ = μ + L_i_ + P_j_ + (LP)_ij_ + e_ijk_, where μ is the overall mean, L_i_ is the fixed effect of LPS (*i* = 1–2 for dosage), P_j_ is the fixed effect of pH (*j* = 1–2), (LP)_ij_ is the fixed effect of the LPS treatment × pH, and e_ijk_ is the random residual error. Differences with *P* < 0.05 were considered significant.

## Results

### Ruminal pH, SCFA and LPS

The results presented here must be interpreted in light of the overall effects of HC feeding on rumen fermentation in the experimental cattle reported in Mao’s prior study [15]. Briefly, the ruminal pH of the HC-fed cattle was lower (*P* < 0.001) than that of the LC-fed cattle, and the duration of time for which the ruminal pH was less than 5.8 was about 5.1 h after the first feeding in the HC group. The HC feeding increased (*P* < 0.001 to *P* = 0.018) the concentrations of propionate (24.32 mmol/L vs. 20.39 mmol/L), butyrate (14.39 mmol/L vs. 11.28 mmol/L), valerate (7.27 mmol/L vs. 4.81 mmol/L), isovalerate (2.64 mmol/L vs. 2.08 mmol/L), total volatile fatty acid (124.85 mmol/L vs. 111.23 mmol/L), lactic acid (0.24 mmol/L vs. 0.18 mmol/L) and LPS (26,266.88 EU/mL vs. 14,741.13 EU/mL) compared with the LC group.

### Blood chemistry and hematology parameters

Compared with LC group, HC feeding led to an increase (*P* < 0.05) in white blood cells (WBC) and lymphocytes (Table 2), whereas HC treatments had no significant effects (*P* > 0.05) on the number of neutrophils and monocytes in blood. Concentrations of serum total protein, globulin, cholesterol and low density lipoprotein were lesser (*P* < 0.05) and the albumin concentration were greater (*P* < 0.05) in the HC group compared with the LC group. There were no differences (*P* > 0.05) in the urea nitrogen, glucose triglyceride, and high density lipoprotein between the two treatments.

**Table 2:** Mean values for blood chemistry and hematology for cows fed two different diets

Item	Diet			*P*-value		
	LC^a^	HC^b^	SEM	Diet	Day	Diet x Day
White blood cells, 10^12^/L	7.30	8.82	0.326	0.004	0.001	0.091
Neutrophils, 10^9^/L	2.27	2.58	0.171	0.212	0.412	0.164
Lymphocytes, 10^9^/L	3.81	4.90	0.242	0.005	<0.001	0.084
Monocytes, 10^9^/L	1.22	1.08	0.075	0.185	0.029	0.295
Total protein, g/L	85.16	78.55	1.537	0.006	0.091	0.484
Globulin, g/L	54.75	46.69	1.280	0.000	0.091	0.513
Albumin, g/L	30.40	31.86	0.350	0.008	0.295	0.306
Urea nitrogen, mmol/L	8.11	7.56	0.276	0.173	0.542	0.215
Glucose, mmol/L	3.48	3.45	0.074	0.804	0.474	0.234
Triglyceride, mmol/L	0.10	0.09	0.003	0.091	0.124	0.110
Cholesterol, mmol/L	2.40	2.04	0.106	0.024	0.594	0.857
Low density lipoprotein, mmol/L	0.39	0.32	0.024	0.045	0.431	0.958
High density lipoprotein, mmol/L	1.15	1.13	0.041	0.757	0.471	0.967

^a^Low-concentrate diet

^b^High-concentrate diet

x means the interaction between two factors

### Annotation of DEGs using Gene Ontology

**Fig. 1:**
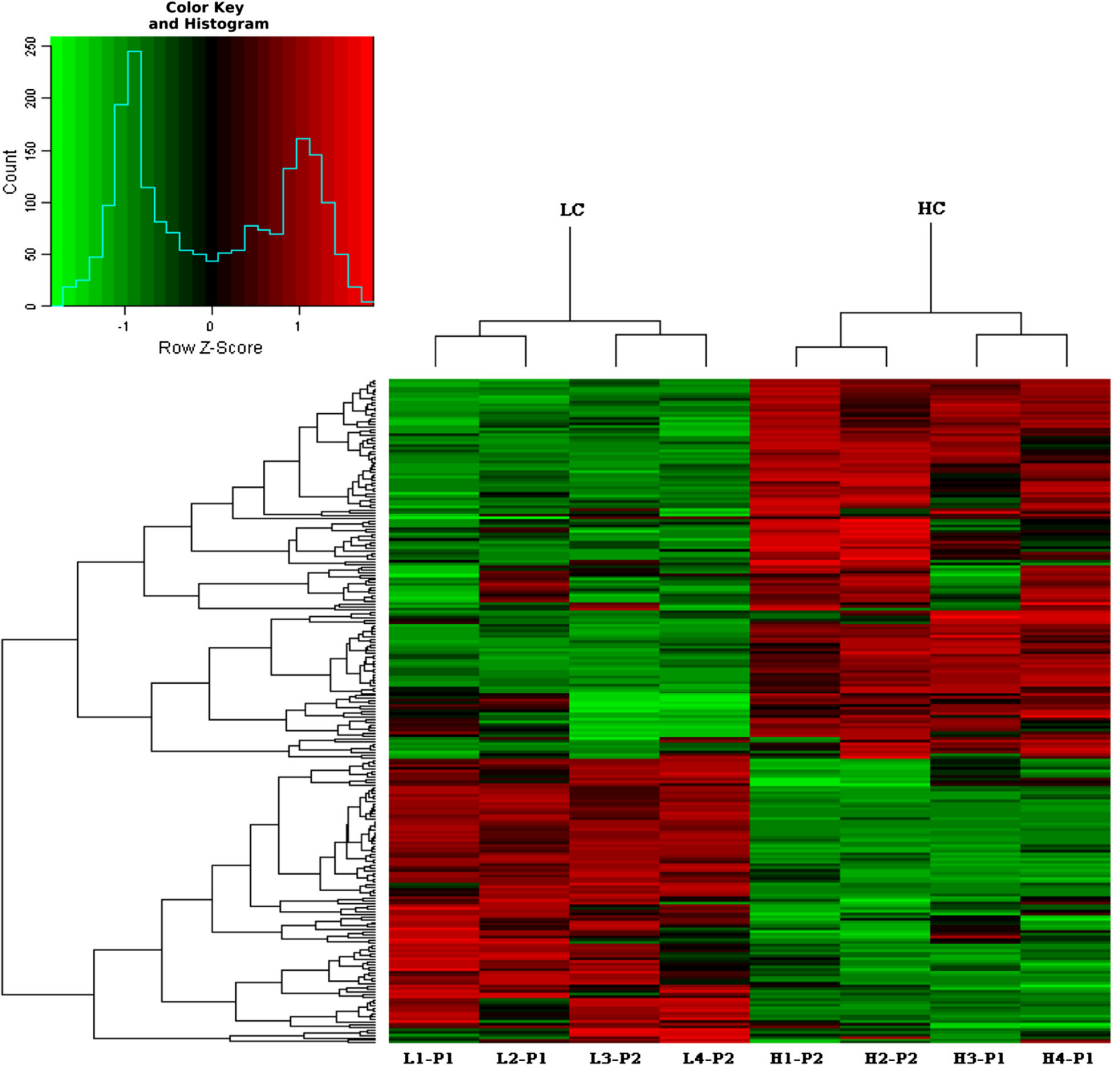
The hierarchical cluster analysis of DEGs. 246 differentially expressed (*P* < 0.05 and fold-change > 1.5) were included in the analysis. The red color indicated the high expression and the green color indicated low expression

**Fig. 2:**
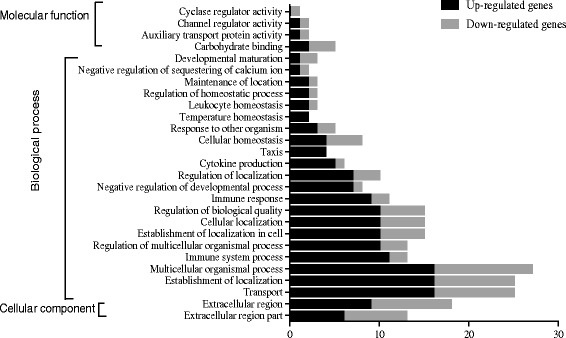
The distribution of DEGs in ontology terms for molecular function, biological process, and cellular component. The black bars indicated the number of up-regulated genes, while the grey bars indicated the number of down-regulated genes

To gain new insights into the underlying biological functions of DEGs, we used GO to analyze the identified DEGs. GO is a well-documented and widely used annotation system that assigns molecular function, biological process, and cellular component information to gene products [22, 23]. A total of 246 DEGs were detected (Additional file 2: Table S2), among them, 141 genes were significantly up-regulated and 105 genes were down-regulated in the HC group compared with the LC group (Fig. 1). Among these DEGs, 87, 77, and 74 DEGs were involved in molecular function, biological process, and cellular component, respectively (Table 3). Within molecular function, DEGs were mainly distributed in categories catalytic activity (31 genes) and binding (67 genes). For further analysis, most proportion of DEGs fell in category protein binding, hydrolase activity, ion binding and nucleic acid binding in GO molecular function. Enrichment analysis indicated category carbohydrate binding, auxiliary transport protein activity, channel regulator activity and cyclase regulator activity within molecular function were affected (*P* < 0.05) by the different diets. Within the cellular component, DEGs were mainly distributed in the categories cell (61 genes) and organelle (33 genes). Only two enriched categories (*P* < 0.05) were found in cellular component, namely extracellular region and extracellular region part. DEGs in the category biological process were mainly distributed in the categories cellular process (65), metabolic process (43 genes), biological regulation (41 genes) and regulation of biological process (37 genes). Our analysis indicated that 24 categories in biological process (at levels 2 and 3) were enriched and included genes associated with the following functions: establishment of localization in cell, immune response, cellular localization, cytokine production, immune system process, etc. (Table 3). The number of genes up- or down- regulated in each GO term and included genes within molecular function, biological process and cellular component is presented in Fig. 2 and Additional file 3: Table S3.

**Table 3:** Enriched GO terms within molecular function, cellular component and biological process

GO ID	Level	Name	Hits	Enrichment test *p*-value	
GO:0030246	3	carbohydrate binding	5	0.026	MF
GO:0015457	2	auxiliary transport protein activity	2	0.046	MF
GO:0016247	3	channel regulator activity	2	0.046	MF
GO:0010851	3	cyclase regulator activity	1	0.045	MF
GO:0044421	2	extracellular region part	13	0.014	CC
GO:0005576	2	extracellular region	18	0.015	CC
GO: 0051649	3	establishment of localization in cell	15	0.001	BP
GO: 0006955	3	immune response	11	0.002	BP
GO: 0051641	3	cellular localization	15	0.003	BP
GO: 0001816	3	cytokine production	6	0.005	BP
GO: 0001776	3	leukocyte homeostasis	3	0.007	BP
GO: 0019725	3	cellular homeostasis	8	0.008	BP
GO: 0032879	3	regulation of localization	10	0.010	BP
GO: 0051239	3	regulation of multicellular organismal process	13	0.011	BP
GO: 0042330	3	taxis	4	0.011	BP
GO: 0002376	2	immune system process	13	0.012	BP
GO: 0051283	3	negative regulation of sequestering of calcium ion	2	0.016	BP
GO: 0032844	3	regulation of homeostatic process	3	0.025	BP
GO: 0001659	3	temperature homeostasis	2	0.028	BP
GO: 0051093	3	negative regulation of developmental process	8	0.038	BP
GO: 0006810	3	transport	25	0.040	BP
GO: 0051707	3	response to other organism	5	0.041	BP
GO: 0051235	3	maintenance of location	3	0.041	BP
GO: 0032501	2	multicellular organismal process	27	0.042	BP
GO: 0051234	2	establishment of localization	25	0.043	BP
GO: 0021700	3	developmental maturation	3	0.044	BP
GO: 0065008	3	regulation of biological quality	15	0.047	BP

*MF* Molecular Function, *CC* Cellular Component, *BP* Biological process

### Assignments of DEGs based on KEGG

**Fig. 3:**
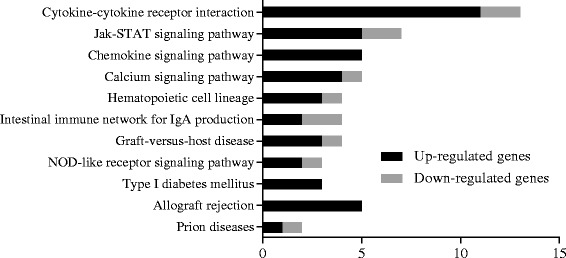
The distribution of DEGs in pathways, only enriched pathways were presented. The black bars indicated the number of up-regulated genes, while the grey bars indicated the number of down-regulated genes

For increased understanding of the effects of the HC diet on rumen tissue functional changes, the identified DEGs were subjected to pathway analysis (Additional file 4: Table S4). Combined with enrichment analysis, 11 KEGG pathways were enriched (*P* < 0.05) by the diets (Fig. 3), including cytokine-cytokine receptor interaction (bta04060), graft-versus-host disease (bta05332), Jak-STAT signaling pathway (bta04630), intestinal immune network for IgA production (bta04672), type I diabetes mellitus (bta04940), hematopoietic cell lineage (bta04640), NOD-like receptor signaling pathway (bta04621), calcium signaling pathway (bta04020), chemokine signaling pathway (bta04062), prion diseases (bta05020) and allograft rejection (bta05330). The number of up- or down- regulated DEGs involved in each KEGG pathway are presented in Fig. 3.

### Functions of inflammatory cytokines in response to ruminal environment variations

Since cytokines always mediated inflammatory responses, the cytokine-cytokine receptor interaction pathway attracted our attention. Of the 13 DEGs involved in this pathway, 11 genes (such as *IL-1β*, *IL-2*, *CCL19*, *CCL8*, *CX3CR1*, *CXCL6*, *IL-22*, *INHBE*, *LEPR*, *PRL*, and *TNFRSF9*) were significantly up-regulated, and only two genes (*IL-6* and *IL15RA*) were significantly down-regulated in the HC group compared with the LC group. For further analysis, these 13 DEGs were classified as multi-functional genes that involved in more than two enriched KEGG pathways or GO terms (Table 4). It is worth noting that the inflammation-related genes *IL-1β*, *IL-2*, and *IL-6* were participated five, seven, and eight pathways, respectively. The three genes were simultaneously involved in four pathways or GO terms, namely graft-versus-host disease (bta05332), extracellular region (GO: 0005576), immune system process (GO: 0002376) and multicellular organismal process (GO: 0032501). Due to the importance of multi-functional genes, the expression of *IL-1β* (up-regulated), *IL-2* (up-regulated) and *IL-6* (down-regulated) was validated by qRT- PCR (Table 5).

**Table 4:**
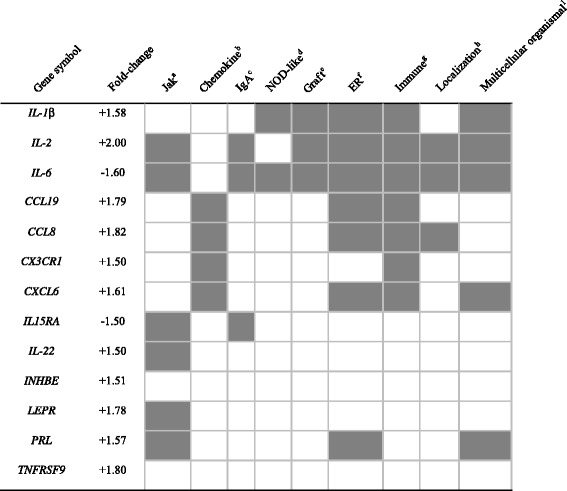
Relationships between DEGs involved in Cytokine-cytokine receptor interaction and other enriched pathways

The grey indicated that this gene was also included in this pathway

^a^Jak-STAT signaling pathway (bta04630)

^b^Chemokine signaling pathway (bta04062)

^c^Intestinal immune network for IgA production (bta04672)

^d^NOD-like receptor (bta04621)

^e^Graft-versus-host disease (bta05332)

^f^Extracellular region part (GO: 0044421)

^g^Immune system process (GO: 0002376)

^h^Establishment of localization (GO: 0051234)

^i^Multicellular organismal (GO: 0032501)

**Table 5:** Validation of microarray gene expressions (*IL-1β*, *IL-2* and *IL-6*) using Realtime-PCR method

Gene symbol	qRT-PCR relative expression			Microarray expression	
	LC	HC	*P*-value	fold change	*P* value
*IL-1β*	1.15 ± 0.164	2.40 ± 0.159	*P* < 0.05	+1.58	*P* < 0.05
*IL-2*	0.80 ± 0.142	1.58 ± 0.135	*P* < 0.05	+2.20	*P* < 0.05
*IL-6*	1.31 ± 0.010	0.66 ± 0.068	*P* < 0.05	- 1.76	*P* < 0.05

### The relationship between the ruminal pH, LPS levels, and cytokines mRNA expression level

**Fig. 4:**
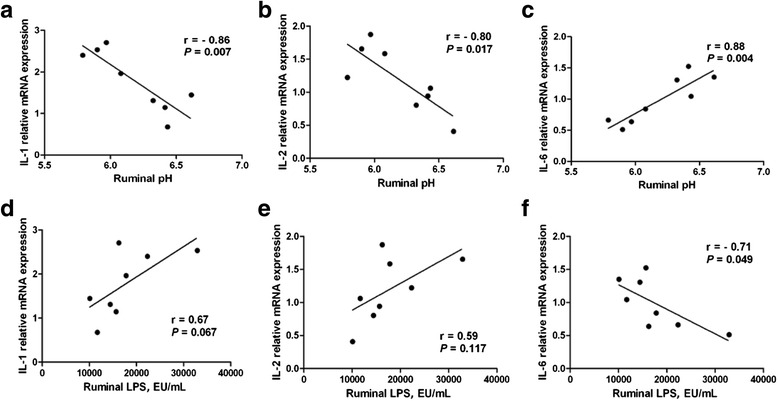
Correlations between cytokine mRNA expression (derived from Realtime-PCR) and ruminal factors. **a**-**c**: Correlation analysis between *IL-1*, *IL-2*, *IL-6* relative mRNA expressions and ruminal pH. **d**-**f**: Correlation analysis between *IL-1*, *IL-2*, *IL-6* relative mRNA expressions and ruminal LPS

To explore whether there were possible relationships between the cytokines’ mRNA expression level and ruminal factors (pH and LPS), correlation analysis was conducted (Fig. 4). Results revealed that ruminal pH exhibited a positive correlation (*P* < 0.05) with *IL-1β* and *IL-2* mRNA expressions but a negative correlation (*P* < 0.05) with *IL-6* mRNA expression. There was a trend toward correlation (*P* = 0.067) and a negative correlation (*P* < 0.05) between the concentration of ruminal LPS and *IL-1β*, *IL-6* relative mRNA expression, respectively. However, no significant correlation between ruminal LPS and *IL-2* mRNA expression was observed.

### Effects of pH and LPS on selected cytokines’ mRNA expression in cultured ruminal epithelial cells

As shown in Table 6, the effects of pH and LPS on these pro-inflammatory mediators in vitro appear to be selective. There was no significant interaction (*P* < 0.05) in the expression of *IL-1β*, *IL-2*, *IL-6*, *IL-8*, *IL-12* and *TNF-α* between LPS and pH. Compared with the control group (group pH = 7.4), the mRNA expression of *IL-1β*, *IL-2*, *IL-6*, and *IL-8* increased (*P* < 0.05) after the LPS treatment. However, the mRNA expression of *IL-12* and *TNF-α* in the cultured ruminal epithelial cells remained unchanged (*P* > 0.05) between the control and LPS treatment groups. Low-pH treatment significantly elevated (*P* < 0.05) the mRNA expression of *TNF-α*, and had no significant effects (*P* > 0.05) on the mRNA expression of *IL-1β*, *IL-2*, *IL-6* and *IL-8* compared with the control group.

**Table 6:** The relative expression of cytokines after low pH and lipopolysaccharides treatments in vitro

Genes	pH 7.4		pH 5.5		SEM	*P* value		
	0	LPS, 10 μg/mL	0	LPS, 10 μg/mL		pH	LPS	pH x LPS
*IL-1β*	1.13	2.75	0.62	2.36	0.259	0.104	0.001	0.103
*IL-2*	1.08	2.06	0.81	2.91	0.280	0.357	0.001	0.093
*IL-6*	0.91	1.66	1.43	1.62	0.106	0.107	0.007	0.068
*IL-8*	0.89	4.34	1.46	3.23	0.455	0.551	<0.001	0.088
*IL-12*	1.04	1.25	0.65	0.94	0.115	0.153	0.292	0.847
*TNF*	0.87	1.62	2.04	2.57	0.255	0.031	0.155	0.783

x which means the interaction between two factors

## Discussion

The overall goal of this study was to characterize the profile of gene expression especially target genes associated with inflammation after SARA challenge in vivo, and to ascertain to find out how environmental factors in rumen affect the mRNA expression of these cytokines in vitro. Therefore, defining SARA was imperative. The ruminal pH, SCFA, and LPS data have been reported previously by our team [15], and the duration time for ruminal pH below 5.8 was about 5.1 h in the HC group during the diurnal period, and the a higher LPS level was observed in the HC group, suggesting that SARA was successfully induced in cows fed the HC diet. Many studies have confirmed that the grain based-SARA challenge can cause translocation of lipopolysaccharide and triggers systemic inflammation leading to an increase in acute phase proteins in blood [7, 24, 25]. In the present study, we cannot absolutely certify the occurrence of systemic inflammation in cows fed HC diet in the absence of detecting acute phase proteins or inflammatory cytokines in blood, as our research centred on local inflammation of the rumen epithelium. However, the increase in the serum white blood cells, lymphocytes, and albumin and the decrease in total protein and globulin in HC-fed cows, indicating that the inflammatory responses may be activated and the synthesis ability of globulin and albumin in liver were affected by HC feeding [26, 27].

In the present study, the analyses of GO categories revealed that most changes centralized in biological processes. In this category, our study showed that GO terms transport, establishment of localization, and cellular localization were enriched by the HC feeding, and these terms were defined as the process or result of the movement of a cell, substance or cellular entity, such as a protein complex or organelle, to a specific location. Considerable evidence has accumulated describing the changes in ruminal epithelial nutrient and ion transport (VFA, glucose, Na^+^), as well as the gene expression of transporters regulating these processes during the adaptation of HC diets [28]. Moreover, HC feeding also induced accelerated differentiation and cellular migration in the stratum basale, spinosum and granulosum layers of the rumen epithelium [14]. It is well established that rumen fermentation profile altered dramatically in response to highly fermentable diets; therefore the transports of these substances and cellular migrations may be driven or stimulated by ruminal fermentation products, and the exact mechanism warrants further exploration. In addition, our study also showed that GO term immune system process and its child terms leukocyte homeostasis and immune response were enriched by the different diet treatments. Indeed, several studies have confirmed that immune system is involved in the adaptation of the rumen epithelium to HC diets, and these immune responses are thought to be initiated from the stimulation of ruminal microbiota or toxin compounds [29, 30]. Correspondingly, the present study demonstrated that the level of some harmful or pro-inflammatory compounds such as LPS, biogenic amines, ethanolamine and glutaric acid in rumen were elevated in the cows fed HC diet (data not published). It is noticeable that a certain degree of immune response may be the self-protective behavior which prevents the further ruminal epithelial injury, and thus, future studies should evaluate the relationship between the degree of immune response and the severity of ruminal acidosis, as well as the process of local inflammation in the rumen epithelium.

To date, relatively little information is available about the global effects of HC feeding on the molecular functions and pathways of the ruminal epithelium. However, a recent study revealed that the alteration of multiple pathways including calcium signaling pathway, gap junction, and Jak-STAT signaling pathway contributes to the changes in ruminal fermentation induced by HC diets [31]. Consistent with the above report, calcium signaling pathway and Jak-STAT signaling pathway were both found enriched in the present study. Enriched calcium pathway in the present study also supported and confirmed the previously observed physiological change that HC feeding clearly altered calcium transport [32], and this alteration in the rumen epithelium may be associated with the stimulation of accumulated VFA in the rumen [33]. Thus, some calcium-dependent events occurred in the rumen epithelium, such as cytokine release and signal transduction process may be affected when cows were fed HC diets. Jak-STAT pathway is one of a handful of pleiotropic cascades used to transduce a multitude of signals for development and homeostasis in mammals [34]. Jak-STAT signaling pathway was reported to be closely related with the activation of inflammation, and the activate JAK and STAT proteins usually played a positive role in inflammation progresses [35]. In the present study, even though the key genes encoded JAK kinases (JAK1, JAK2, JAK3 and Tyk2) and STATs were not changed, enriched Jak-STAT signaling pathway may to some extent reflect that the functional homeostasis of rumen epithelium were impaired after HC feeding.

In the present study, aside from the changes in Calcium signaling pathway and Jak-STAT signaling pathway, inflammation related pathways including Cytokine-cytokine receptor interaction and Chemokine signaling pathway were also enriched by the different diet treatments. Inflammation is a complex set of synergistic actions of inflammatory mediators (such as cytokines, chemokines, and nitric oxide) and immune cells (such as macrophages, lymphocytes, and monocytes), as the first response of the immune system to infection or tissue injury [36, 37]. Although some review articles reported HC feeding may lead to rumenitis [3, 38] local inflammation in the rumen epithelium, there is still a lack of understanding of how inflammatory-related genes respond to HC diet at the molecular level. Our study revealed that the expression of genes found in cytokine-cytokine receptor pathway, including *IL-1β*, *IL-2*, *IL-22*, *CCL19*, *CCL8*, *CX3CR1*, *CXCL6*, *INHBE*, *LEPR*, and *PRL*, were up-regulated in HC-fed cows; only *IL-6* and *IL-15RA* were down-regulated. It was well known that *IL-1β*, *IL-2* and *IL-22* have the potential to stimulate pro-inflammatory responses, and mediated inflammatory diseases [39]. Therefore, the higher expression of *IL-1β*, *IL-2* and *IL-22* mRNA in the rumen epithelium indicates that local inflammation might occur in the rumen epithelium of the HC group. Chemokines are chemotactic cytokines that can participate in regulating inflammatory leukocyte recruitment, lymphocyte recruitment and homing [40]. Chemokines were categorized as inflammatory or homeostatic chemokine according to the functional role. Results from previous studies indicated that bovine mammary epithelial cells can express *CXCL6* (also called *GCP2*) and *CCL8* (also called *MCP2*) in response to certain bacterial cell components during the simulation course of mastitis in vitro [41, 42]. In addition, CX3CR1, the receptor of chemokine CX3CL1, was also classified as an inflammatory chemokine [43]. In the present study, up-regulated genes (*CCL19*, *CCL8*, and *CX3CR1*) were observed in HC-fed cows, further indicating that local inflammation in the rumen epithelium might be triggered.

Inflammation may be the interaction result of multiple pathways or biological processes. In this study, the GO term immune system process was enriched in the HC group, and most affected inflammatory cytokines such as IL-1β, IL-2 and IL-6 were also in the category immune system process, indicating that the immune system activation might be involved in the adaptation of the rumen epithelium to the HC diet. Of the affected pro-inflammatory cytokines, there is a paucity of information on the expression of IL-1β in ruminants fed HC diet. However, IL-1β has been shown to increase the permeability of the intestinal tight junctions of Caco-2 cells [44]. If IL-1β acts similarly in the rumen epithelium, the interleukin may play a role in reducing the barrier function of the rumen epithelium in cattle fed the HC diet. Previous studies showed that inducers of *IL-1β* expression in the gut include hyperosmolarity, T and B cells, LPS, and other interleukin proteins [44, 45] indicating that these factors may have played a role in IL-1β up-regulation in cattle fed high grain diets. This is also consistent with our in vitro results that LPS treatment significantly increased the expression of *IL-1β*. The role of IL-2 in the inflammatory process is complex and involves pro-inflammatory as well as regulatory aspects, and dysregulation seem to contribute to various immune system-related diseases in humans [46, 47]. A previous study showed that the aberrant production of IL-2 can disrupt primary human T lymphocytes function by leading to the loss of T cell anergy and induction of autoimmunity [48]. Thus, it is reasonable to postulate that an increase in the expression of *IL-2* during HC feeding might have a negative impact on the innate immune response in the rumen epithelium. IL-6 is a multifunctional, pleiotropic cytokine involved in regulating immune responses, acute-phase responses and inflammation [49, 50]. Previous studies revealed that IL-6 is involved in tissue repair and cytoprotection in the human gut, and preventing IL-6 production could favor wound healing and remodelling following injury [39, 50, 51]. In this study, the true cause of the observed effects in the down-regulation of *IL-6* mRNA in the HC group is unclear. Nevertheless, since the reduction of pro-inflammatory cytokines such as IL-1β, IL-6, and TNF-α can reduce the length and severity of the immune response [52], it follows that decreasing the expression of IL-6 during HC feeding can have beneficial effects.

To validate the results of the in vivo experiment and get a better understanding of the inflammation response during HC feeding, the rumen factors (LPS and low pH) which are most associated with SARA were selected for the in vitro experiment. In vitro, the mRNA expression of tested pro-inflammatory mediators *IL-1β*, *IL-2*, and *IL-8* was up-regulated after LPS was stimulated. These results further strengthened the well-documented theory of the pro-inflammation ability of LPS, and demonstrated that the higher levels of LPS in rumen fluid during HC feeding could contribute to local inflammation in the rumen epithelium. Our in vitro results also showed that the expression of *IL-6* mRNA was increased by the LPS challenge, and this is not consistent with the down-regulation results observed in vivo in the present study. These differences indicate that *IL-6* mRNA expression might be affected by factors other than LPS. Indeed, recent progress in the understanding of the role butyrate plays in inflammation confirmed that n-butyrate can down-regulate the level of IL-6 during LPS-induced inflammation response but not affect the level of TNF-α [41]. In this study, a higher concentration of butyrate was observed in the HC group compared with the LC group. Therefore, the stimulation effect of LPS on the production of IL-6 might be concealed by the inhibition effect of butyrate, and this might finally result in the lower mRNA expression of *IL-6* observed in the in vivo tests.

Although it has been well known that feeding dairy cows diets containing high proportions of grain is associated with a rapid decline in ruminal pH, and low ruminal pH can lead to rumenitis and eventually to ruminal parakeratosis, erosion, and ulceration of the rumen epithelium [30], there is little information on the effects of extracellular pH on rumen immune function. Clinical studies of organic acidosis in humans revealed clinical acidaemias are usually accompanied by immune deficiency, including a decrease in white cell numbers, globulins, and mitogenic responses, a diminution of the inflammatory response and delayed phagocytosis [53]. In this study, our findings revealed that low-pH treatment significantly elevated the mRNA expression of gene *TNF-α* compared with the high pH group. These results suggest that lower pH may contribute to local inflammation of the rumen epithelium by up-regulating certain genes. However, the mechanism underlying the up-regulation of *TNF-α* expression induced by lower pH during HC feeding is unclear, and more research is needed to evaluate the effects of extracellular pH on the immunity function of the rumen epithelium.

## Conclusions

Our findings revealed that the biological functional changes in the rumen epithelium were mainly focused on biological processes in dairy cattle fed a high-concentrate diet, and the local inflammation response was triggered during HC feeding in the present study. The lower pH and high level of LPS in rumen during HC feeding may play an important role in the process of local inflammation in the rumen epithelium, and their pro-inflammatory effects may weaken the permeability barrier of the rumen epithelium. Future research is required to explore the mechanism of rumen epithelium inflammation as well as the role of lower ruminal pH in the damage to the rumen tissue of dairy cattle during SARA.

## Abbreviations

DEGs, differentially expressed genes; HC, high-concentrate; Hp, haptoglobin; LC, low-concentrate; SAA, serum amyloid A; SARA, subacute ruminal acidosis; TMR, a total mixed ration; VFA, volatile fatty acids

## Additional files

Additional file 1: Table S1. Ingredients and chemical composition of diets. (DOCX 17 kb) Additional file 2: Table S2. List of differentially expressed genes. (PDF 254 kb) Additional file 3: Table S3. Enriched GO terms (only significantly enriched GO terms were listed). (PDF 20 kb) Additional file 4: Table S4. DEGs were assigned to KEGG pathway. (PDF 29 kb)
