# Supplementary material for: High-concentrate feeding upregulates the expression of inflammation-related genes in the ruminal epithelium of dairy cattle
Source: J Anim Sci Biotechnol. 2016 Jul 29;7:42. doi: 10.1186/s40104-016-0100-1 (PMC4966727; doi:10.1186/s40104-016-0100-1)
Supplement: Additional file 1: Table S1. — Ingredients and chemical composition of diets. (DOCX 17 kb) [file 40104_2016_100_MOESM1_ESM.docx]

Table S1 Ingredients and chemical composition of diets.

| Ingredients (% of DM)^a^ | LC | HC |
| --- | --- | --- |
| Alfalfa hay | 27.00 | 12.00 |
| Leymus chinensis | 3.00 | 4.00 |
| Straw | 0.00 | 4.00 |
| Maize silage | 30.00 | 10.00 |
| Maize | 20.00 | 45.00 |
| Wheat bran | 0.00 | 1.40 |
| Soybean meal | 10.85 | 15.00 |
| cottonseed meal | 6.00 | 5.00 |
| Calcium carbonate | 0.40 | 0.85 |
| Calcium hydrogen phosphate | 0.75 | 0.75 |
| Sodium bicarbonate | 0.75 | 0.75 |
| Sodium chloride | 0.50 | 0.50 |
| Dairy premix^b^ | 0.75 | 0.75 |
| Nutrient composition (% of DM) | | |
| NEL(Mcal kg^-1^ of DM) | 1.54 | 1.71 |
| Crude protein (CP) | 15.8 | 15.8 |
| Neutral detergent fiber (NDF) | 34.9 | 24.7 |
| Acid detergent fiber | 23.0 | 13.2 |
| Non-fiber carbohydrates (NFC) | 36.1 | 46.3 |
| Calcium | 0.88 | 0.82 |
| Phosphorous  Starch^c^ | 0.51  13.57 | 0.53  30.36 |

^a^ Fed as TMR.

^b^ Contained Na, 0.24%; K, 0.5%; S, 0.2%; Zn, 4,000 mg/kg; Cu, 1,000 mg/kg; Mn, 2,500 mg/kg; I, 64 mg/kg; Co, 5 mg/kg; vitamin A, 1,000,000 IU/kg; vitamin D, 110,000 IU/kg, and vitamin E, 6000,000 IU/kg.

LC= low concentrate diet, HC= high concentrate diet

^c^Not determined, values were calculated
