# Supplementary material for: High-concentrate feeding upregulates the expression of inflammation-related genes in the ruminal epithelium of dairy cattle
Source: J Anim Sci Biotechnol. 2016 Jul 29;7:42. doi: 10.1186/s40104-016-0100-1 (PMC4966727; doi:10.1186/s40104-016-0100-1)
Supplement: Additional file 2: Table S2. — List of differentially expressed genes. (PDF 254 kb) [file 40104_2016_100_MOESM2_ESM.pdf]

Table S2 List of differentially expressed genes

GeneBankAcc	Symbol	*P* value	FC	Description
XM_590017	LOC512493	0.048	-1.70	similar to KIAA2022 protein
XM_865303	LOC614010	0.047	-2.13	PREDICTED: Bos taurus similar to fatty acid desaturase 2
EE959717	K40110A	0.005	-1.75	FNM Bos taurus cDNA clone K4011 5-
XM_002692067	IL15RA	0.026	-1.50	interleukin 15 receptor, alpha
NM_001025332	FABP2	0.029	-2.31	fatty acid binding protein 2, intestinal
NM_175715	ADCYAP1R1	0.034	-1.50	adenylate cyclase activating polypeptide 1 receptor type I
XM_616648	TDRD1	0.024	1.73	tudor domain containing 1
NM_001034252	FBXL21	0.002	-1.58	Bos taurus F-box and leucine-rich repeat protein 21
EE892165	A79630A	0.022	2.93	FFB Bos taurus cDNA clone A7963 5-
XM_00178926(	ROR1	0.022	1.61	receptor tyrosine kinase-like orphan receptor 1
NM_00102534{	SPINK1	0.010	-2.08	serine peptidase inhibitor, Kazal type 1
NM_00120638(	SLC12A5	0.010	-2.37	solute carrier family 12, member 5
NM_00110209{	CCDC85A	0.015	2.22	coiled-coil domain containing 85A
NM_00119281(	LGI2	0.011	-1.77	leucine-rich repeat LGI family, member 2
XM_866198	LOC614634	0.030	-1.70	similar to glutamate receptor delta-1 subunit
NM_00110197{	TMPRSS4	0.050	1.55	transmembrane protease, serine 4
XM_604089	GPR176	0.009	1.76	G protein-coupled receptor 176
NM_00107806(	DMRT1	0.003	-2.20	doublesex and mab-3 related transcription factor 1
CB433840		0.045	-1.67	MARC 6BOV Bos taurus cDNA 3-
NM_00110219(	RIMS2	0.023	1.65	regulating synaptic membrane exocytosis 2
NM_00119287{	HOXC11	0.008	-7.83	homeobox C11
XM_581197	TSC2	0.018	-2.39	similar to tuberous sclerosis 2
BI538290	CPLX2	0.037	-2.17	MARC 4BOV Bos taurus cDNA 5-
CB423555		0.013	-1.50	MARC 6BOV Bos taurus cDNA 5-
BC134447	TP53RK	0.021	1.50	TP53 regulating kinase
XM_00269801(	ACSM4	0.042	-2.27	acyl-CoA synthetase medium-chain family member 4
XM_002688104	LOC511618	0.021	2.77	similar to rCG28550
NM_001075662	SDS	0.049	4.74	serine dehydratase
NM_001076092	DZIP3	0.043	1.54	DAZ interacting protein 3, zinc finger

BI848985	ALOX15	0.043	1.54	MARC 2BOV Bos taurus cDNA 5-
NM_00107630	COLEC11	0.046	-1.53	collectin sub-family member 11
EE911885	A47701A	0.029	-1.66	FFB Bos taurus cDNA clone A4770 3-
NM_00120618	SEMA7A	0.050	1.54	semaphorin 7A, GPI membrane anchor
NM_174177	SCIN	0.040	1.50	scinderin
XM_601937	LOC523635	0.032	1.52	similar to UPF0183 protein
NM_174786	GUCA1B	0.000	-2.16	guanylate cyclase activator 1B (retina)
EU581635	FIGLA	0.003	1.83	folliculogenesis specific basic helix-loop-helix
NM_00109807	SCNN1B	0.045	1.51	sodium channel, nonvoltage-gated 1, beta
NM_00108036	MGC139448	0.044	1.92	hypothetical protein LOC787473
NM_174446	RAB3A	0.013	-1.78	RAB3A, member RAS oncogene family
NM_001079771	SMOC1	0.015	-2.15	SPARC related modular calcium binding 1
NM_00120591	SMG1	0.020	1.62	smg-1 homolog, phosphatidylinositol 3-kinase-related kinase
XM_600422	LOC522147	0.050	-1.61	similar to obscurin, cytoskeletal calmodulin and titin-interacting RhoGEF
NM_00108371	MYLK2	0.045	1.72	myosin light chain kinase 2
NM_00101568	NAPEPLD	0.026	1.61	N-acyl phosphatidylethanolamine phospholipase D
NM_00119137	KCND3	0.044	-1.75	potassium voltage-gated channel, Shal-related subfamily, member 3
CB535104	768488	0.002	1.92	MARC 6BOV Bos taurus cDNA 3-
NM_174292	CRYGS	0.015	1.51	crystallin, gamma
NM_00107574	GBP5	0.043	1.50	guanylate binding protein 5
ENSBTAT0000	TCN1	0.047	3.15	transcobalamin I (vitamin B12 binding protein, R binder family)
XM_580797	MUC20	0.034	1.54	mucin 20, cell surface associated
XM_870023	EFNB3	0.020	2.49	ephrin-B3
XM_00268666	DLX6	0.035	1.86	distal-less homeobox 6
XM_612284	TTC21A	0.046	-1.50	tetratricopeptide repeat domain 21A
XM_592866	HTRA4	0.027	1.85	HtrA serine peptidase 4
XM_866835	LOC615115	0.026	-1.68	similar to OTTHUMP00000016363
NM_00120625	CRISP1	0.050	-1.61	cysteine-rich secretory protein 1
XM_591914	SFT2D3	0.042	2.56	SFT2 domain containing 3
XM_602209	LOC523898	0.021	-1.60	similar to sulfotransferase K1
XM_617812	SYT6	0.026	1.66	synaptotagmin VI
NM_00120584	PDILT	0.017	-1.79	protein disulfide isomerase-like, testis expressed

NM_001101222	GDAP1	0.047	1.50	ganglioside-induced differentiation-associated protein 1
NM_001192534	MKX	0.042	-2.04	mohawk homeobox
NM_001113762	ICAM5	0.039	1.50	intercellular adhesion molecule 5, telencephalin
NM_001198991	CR2	0.006	1.76	complement component (3d/Epstein Barr virus) receptor 2
XM_594203	LOC516067	0.026	-2.73	similar to folate receptor 1 precursor, transcript variant 1
XM_614208	LOC534441	0.035	-1.71	similar to Huntingtin interacting protein K, transcript variant 1
NM_001075523	KRT25	0.014	-1.95	keratin 25
XM_586585	FRMPD4	0.014	-1.92	FERM and PDZ domain containing 4
NM_001102558	CX3CR1	0.042	1.50	chemokine (C-X3-C motif) receptor 1
CB451845		0.003	-1.55	MARC 6BOV Bos taurus cDNA 3-
NM_001038682	AICDA	0.016	-2.35	activation-induced cytidine deaminase
NM_174300	CXCL6	0.050	1.61	chemokine (C-X-C motif) ligand 6
NM_001078017	LOC538872	0.020	-1.59	similar to Dual specificity protein phosphatase 18
NM_001034345	CCL19	0.042	1.79	chemokine (C-C motif) ligand 19
NM_001080738	LRRN1	0.019	-1.67	leucine rich repeat neuronal 1
NM_001001145	BSP1	0.026	-1.81	binder of sperm 1
XM_865022	CEP112	0.023	-1.71	coiled-coil domain containing 46
CR851521		0.040	2.17	Normalized and Subtracted endometrium tissues cDNA clone
XM_864903	LOC613791	0.038	-2.41	similar to pregnancy-associated glycoprotein-18
NM_001075545	DHRS7C	0.050	-1.51	dehydrogenase/reductase (SDR family) member 7C
NM_001012678	BOLA-DYA	0.001	2.14	major histocompatibility complex, class II, DY alpha
NM_001206235	LOC616136	0.024	1.50	hypothetical LOC616136
XM_609180	ATP12A	0.017	3.66	ATPase, H+/K+ transporting, nongastric, alpha polypeptide
XM_880264	LOC527819	0.048	2.07	similar to keratin 4, transcript variant 3
XM_588578	LOC539654	0.023	1.64	similar to protocadherin gamma subfamily A, 2
XM_585407	LOC508610	0.002	-2.20	similar to olfactory receptor, family 4, subfamily C, member 45
XM_869779	LOC617506	0.003	1.81	similar to homeo box (H6 family) 2
NM_001083745	GTF2IRD2	0.042	1.92	GTF2I repeat domain containing 2
AW658518		0.014	1.97	MARC 1BOV Bos taurus cDNA 5-
XM_001789474	LOC526286	0.009	-1.57	similar to olfactory receptor MOR223-1
BI681020		0.011	1.93	MARC 1BOV Bos taurus cDNA 5-, mRNA sequence
NM_173923	IL6	0.026	-1.60	interleukin 6 (interferon, beta 2)

XM_589391	TNIP3	0.022	4.98	similar to TNFAIP3 interacting protein 3
XM_00178855{8}	THOC1	0.032	2.21	THO complex 1
NM_174386	MFAP5	0.045	1.82	Bos taurus microfibrillar associated protein 5
DV880987	FUT11	0.020	-1.63	fucosyltransferase 11 (alpha (1,3) fucosyltransferase)
XM_596187	LOC518005	0.018	2.20	similar to NM23-H8
XM_866469	LOC614829	0.039	-1.87	similar to AMP-activated protein kinase alpha 2 catalytic subunit
XM_870215	LOC617885	0.041	1.57	similar to amyloid beta A4 precursor protein-binding, family A, member 1
XM_002697311	PKHD1	0.017	-2.08	polycystic kidney and hepatic disease 1 , transcript variant X3
NM_00103361(	KRT8	0.014	-1.55	keratin 8
XM_606357	LOC527950	0.025	-1.73	similar to potassium voltage-gated channel, subfamily H , member 7
XM_867323	CD226	0.036	1.77	CD226 molecule, transcript variant X1
XM_591375	LOC540082	0.024	1.51	similar to olfactory receptor, family 1, subfamily A, member 1
NM_00107687(	IHH	0.042	-1.66	Indian hedgehog homolog (Drosophila)
NM_00103459(	MGC128811	0.032	-1.74	chromosome 28 open reading frame, human C10orf58
NM_00120605(	GRIA3	0.014	2.53	glutamate receptor, ionotrophic, AMPA 3
XM_580713	LOC507882	0.050	1.80	similar to mCG53389
NM_001076912	SCN4B	0.042	-1.55	sodium channel, voltage-gated, type IV, beta
EE896477	B02260A	0.016	1.54	FFB Bos taurus cDNA clone B0226 5-
NM_174391	MMP20	0.010	-1.50	matrix metallopeptidase 20
XM_592737	LOC514827	0.049	-1.80	similar to ATP-binding cassette, sub-family C, member 4
NM_001076954	GOLGA7B	0.035	-1.92	golgi autoantigen, golgin subfamily a, 7B
CB417821	SP5	0.044	1.51	Sp5 transcription factor
XM_599713	LOC521449	0.027	2.05	hypothetical protein FLJ32310
XM_590420	ARL14	0.024	1.74	ADP-ribosylation factor-like 14
NM_00101267{5}	BOLA-DQA5	0.017	1.58	major histocompatibility complex, class II, DQ alpha 5
NM_00120596{3}	SYT15	0.021	-2.07	synaptotagmin XV
NM_00119257(	TREH	0.041	-1.66	trehalase (brush-border membrane glycoprotein)
XM_599343	LOC521086	0.019	-1.62	similar to poly(A) binding protein, cytoplasmic 1-like 2B
XM_603860	KCNA3	0.001	1.65	potassium voltage-gated channel, shaker-related subfamily, member 3
NM_001205842	INHBE	0.023	1.51	inhibin, beta E
NM_177517	ST6GAL1	0.014	2.07	ST6 beta-galactosamide alpha-2,6-sialyltranferase 1
NM_00107602(	CADPS	0.006	-1.82	Ca++-dependent secretion activator

XM_612626	LOC533271	0.042	-1.51	similar to glutamate receptor, metabotropic 8
DY168909	SIGLEC12	0.022	1.73	sialic acid binding Ig-like lectin 12
NM_174093	IL1β	0.050	1.58	interleukin 1, beta
NM_176618	PAG7	0.030	-2.07	pregnancy-associated glycoprotein 7, transcript variant 1
NM_00119282[illegible]	RRAGD	0.005	-1.90	Ras-related GTP binding D
XM_865961	LOC614450	0.005	1.63	similar to NOPAR2
XM_586824	HSD11B1	0.021	-1.81	similar to hydroxysteroid (11-beta) dehydrogenase 1
XM_606959	CDK5R2	0.013	-1.50	similar to cyclin-dependent kinase 5, regulatory subunit 2 (p39)
XM_865596	LOC614197	0.036	-1.58	similar to Uncharacterized protein C14orf105
NM_00101228[illegible]	LEPR	0.017	1.78	Bos taurus leptin receptor
NM_00119127[illegible]	KLF12	0.032	1.53	Kruppel-like factor 12
L13938	PLCB4	0.007	1.65	Bovine phospholipase C
XM_580794	HTR7	0.016	-2.21	5-hydroxytryptamine receptor 7
XM_868456	LOC616432	0.021	-1.57	similar to proline rich membrane anchor 1
AW670011	AFF2	0.013	-2.23	similar to AF4/FMR2 family, member 2
NM_00120660[illegible]	LOC524694	0.039	-2.04	similar to Paladin
NM_00103533[illegible]	TNFRSF9	0.048	1.80	tumor necrosis factor receptor superfamily, member 9
NM_174733	DHRS9	0.023	1.53	dehydrogenase/reductase (SDR family) member 9
NM_173953	PRL	0.018	1.57	prolactin
EE970120	Q23660A	0.011	2.76	Q23660A FNM Bos taurus cDNA clone Q2366 5-
NM_00103746[illegible]	MGC133641	0.034	1.56	similar to Homo sapiens histone 1, H2bc
XM_615275	LOC535243	0.029	1.53	similar to cytochrome P450, family 2, subfamily C, polypeptide 18
EE904857	B50401A	0.042	-1.52	B50401A FFB Bos taurus cDNA clone B5040 3-
XM_580533	CHL1	0.033	-8.23	similar to cell adhesion molecule with homology to L1CAM
XM_594382	ASCL1	0.026	-2.08	similar to achaete-scute complex-like 1 (Drosophila)
NM_001105212	KIF17	0.012	-1.70	kinesin family member 17
NM_00104660[illegible]	RGS4	0.027	1.59	regulator of G-protein signaling 4
XM_00358204[illegible]	ABCA13	0.008	-1.65	ATP-binding cassette, sub-family A, member 13
NM_001046064	OIT3	0.005	3.21	oncoprotein induced transcript 3
BC148911	TRDN	0.006	-2.55	triadin
NM_001035107	TINAG	0.025	-1.54	tubulointerstitial nephritis antigen
BC110200	H1FNT	0.013	2.28	H1 histone family, member N, testis-specific

NM_00107783	SFTPA1	0.031	-1.70	surfactant protein A1
NM_00120649	ITGB3	0.016	1.91	integrin, beta 3 (platelet glycoprotein IIIa, antigen CD61)
NM_00107561	TXNDC6	0.027	-1.66	thioredoxin domain containing 6
NM_00119284	SLCO4C1	0.013	1.54	solute carrier organic anion transporter family, member 4C1
NM_174373	KCNJ2	0.005	1.50	potassium inwardly-rectifying channel, subfamily J, member 2
XM_580632	OR2D2	0.041	1.55	olfactory receptor, family 2, subfamily D, member 2
XM_582699	TMEM221	0.030	1.99	transmembrane protein 221
AW430375	SLC38A4	0.036	1.87	solute carrier family 38, member 4
NM_00110229	PTPN5	0.010	1.77	protein tyrosine phosphatase, non-receptor type 5
CB451858	CREB1	0.014	-2.29	cAMP responsive element binding protein 1, transcript variant A
EE944005	G64131A	0.034	1.61	Intronic region in predicted Homo sapiens hypothetical protein FLJ34690
NM_00107563	VAMP1	0.013	-1.70	vesicle-associated membrane protein 1 (synaptobrevin 1)
XM_599201	LOC520949	0.030	1.70	similar to KIAA0416, transcript variant 1
XM_00269644	LOC783755	0.009	1.72	similar to Dynein heavy chain 5, axonemal
NM_00119217	CLSPN	0.004	2.24	claspin
ENSBTAT0000	RYR3	0.039	2.00	ryanodine receptor 3
XM_590977	BCORL1	0.006	-1.50	BCL6 corepressor-like 1
NM_174288	CRTL1	0.029	1.85	cartilage linking protein 1
NM_00109937	SLC15A1	0.040	1.64	solute carrier family 15 (oligopeptide transporter), member 1
XM_602101	ABCB4	0.028	2.12	ATP-binding cassette, sub-family B (MDR/TAP), member 4
NM_00120629	GIPC3	0.002	2.35	GIPC PDZ domain containing family, member 3
XM_864611	SIM1	0.025	-1.64	single-minded homolog 1 (Drosophila)
NM_00110996	DCLK1	0.024	-2.14	doublecortin-like kinase 1
EE910096	A37090A	0.046	2.31	FFB Bos taurus cDNA clone A3709 5-
XM_613195	LHX4	0.035	1.73	LIM homeobox 4
XM_866545	IRX4	0.031	1.96	iroquois homeobox protein 4
NM_00119231	RTKN2	0.007	1.69	rhotekin 2
NM_00108245	WNT11	0.007	-1.80	wingless-type MMTV integration site family, member 11
NM_00100144	LOC407238	0.043	1.81	estrogen receptor alpha
BF606645		0.023	1.68	MARC 3BOV Bos taurus cDNA 5-
CB170988	TRQ6030013	0.035	1.55	CSEQFN22 thyroid and parathyroid
AW325465		0.016	1.85	16151 MARC 4BOV Bos taurus cDNA 5-

NM_00101491	HBE4	0.025	1.99	hemoglobin, beta, epsilon 4
NM_174007	CCL8	0.033	1.82	chemokine (C-C motif) ligand 8
XM_00268724	KRT77	0.021	-1.70	keratin 77
XM_584248	LOC507604	0.001	-2.03	similar to Kinesin-like protein KIF1B (Klp)
NM_00109837	IL22	0.013	1.50	interleukin 22
NM_00110034	AMPD1	0.036	2.12	adenosine monophosphate deaminase 1 (isoform M)
ENSBTAT0000	UGGT2	0.041	1.80	UDP-glucose glycoprotein glucosyltransferase 2
NM_00102450	IRF9	0.044	1.59	interferon regulatory factor 9
XM_587381	LOC510256	0.017	-2.04	similar to SV2 related protein
CB440453		0.025	1.54	MARC 6BOV Bos taurus cDNA 3
XM_602041	LOC523738	0.036	1.50	similar to tripartite motif protein 17
EE908476	A28420A	0.026	-3.14	FFB Bos taurus cDNA clone A2842 5-
NM_00120614	TXK	0.017	1.57	TXK tyrosine kinase
NM_00107790	INSIG1	0.027	-1.50	insulin induced gene 1
XM_600886	LOC522601	0.011	1.50	similar to dentin sialophosphoprotein preproprotein
NM_001076092	DZIP3	0.034	1.57	DAZ interacting protein 3, zinc finger
XM_865632	NEFH	0.038	2.58	neurofilament, heavy polypeptide
NM_001075414	IFIT3	0.003	2.33	interferon-induced protein with tetratricopeptide repeats 3
XM_869498	AMAN	0.050	1.67	similar to Epididymis-specific alpha-mannosidase precursor
XM_592839	LOC540268	0.016	1.72	hypothetical LOC540268
BC133554	LOC614587	0.043	1.62	similar to Uncharacterized protein C10orf90
CB451086		0.008	2.11	MARC 6BOV Bos taurus cDNA 3-
NM_001098461	SLC6A14	0.017	2.69	solute carrier family 6 (amino acid transporter), member 14
XM_00268788	FGF23	0.026	2.56	fibroblast growth factor 23
XM_867744	RBM15B	0.010	-2.20	RNA binding motif protein 15B
CB440543		0.039	-1.71	MARC 6BOV Bos taurus cDNA 5-
XM_612397	LOC540631	0.023	-1.59	similar to kelch-like 23
NM_001033761	TLR7	0.032	1.60	toll-like receptor 7
NM_174654	SLCO1A2	0.002	-1.62	solute carrier organic anion transporter family, member 1A2
XM_582333	LOC505959	0.016	4.48	similar to Potassium voltage-gated channel subfamily H member 8
NM_00104635	SULT2A1	0.003	-1.56	sulfotransferase family, cytosolic, 2A
NM_00101561	PLA1A	0.026	1.81	phospholipase A1 member A

XM_868741	CCDC7	0.009	-1.90	coiled-coil domain containing 7，transcript variant X3
XM_604156	LOC525800	0.041	1.86	hypothetical LOC525800
NM_00107802(	DDI1	0.022	-1.50	DDI1, DNA-damage inducible 1, homolog 1
XM_866470	LOC614830	0.001	1.56	similar to RAB6A, member RAS oncogene family
XM_581305	WNT6	0.042	-1.56	wingless-type MMTV integration site family, member 6
NM_180997	IL2	0.014	2.00	interleukin 2
NM_00119248(	ADAMTS18	0.006	-1.57	ADAM metallopeptidase with thrombospondin type 1 motif, 18
NM_00109890	CDH17	0.019	2.28	cadherin 17, LI cadherin
BC109539	LOC520778	0.023	-1.70	similar to ubiquilin 2
XM_605253	UNC13A	0.021	-2.08	unc-13 homolog A (C. elegans)
XM_00358233(	TMPRSS11B	0.006	5.59	transmembrane protease, serine 11B
NM_174619	STXBP1	0.023	1.61	syntaxin binding protein 1
XM_580501	LOC504388	0.005	1.98	olfactory receptor 1307-like
XM_589867	LOC539857	0.032	2.33	similar to Protocadherin alpha 10 precursor
NM_00120584	CASP14	0.003	1.60	caspase 14, apoptosis-related cysteine peptidase
NM_00107589(	KLK10	0.042	1.68	kallikrein-related peptidase 10
NM_00119222	RRH	0.010	1.95	retinal pigment epithelium-derived rhodopsin homolog
NM_00107602(	CADPS	0.004	-1.82	Ca++-dependent secretion activator
XM_00178853(	LOC787880	0.035	3.42	similar to Synaptotagmin-14 (Synaptotagmin XIV)
NM_00101555(	CDH16	0.005	1.76	cadherin 16, KSP-cadherin
NM_00120686	LOC617781	0.050	1.73	similar to Uncharacterized protein C12orf34
NM_174042	DRD1	0.023	2.98	dopamine receptor D1
XM_581757	LOC505468	0.004	2.02	cytochrome P450 2C19
XM_864633	LOC613645	0.037	-1.56	similar to regulating synaptic membrane exocytosis 2
XM_867850	C3H1orf177	0.003	-1.77	similar to Uncharacterized protein C1orf177
