# Supplementary material for: High-concentrate feeding upregulates the expression of inflammation-related genes in the ruminal epithelium of dairy cattle
Source: J Anim Sci Biotechnol. 2016 Jul 29;7:42. doi: 10.1186/s40104-016-0100-1 (PMC4966727; doi:10.1186/s40104-016-0100-1)
Supplement: Additional file 3: Table S3. — Enriched GO terms (only significantly enriched GO terms were listed). (PDF 20 kb) [file 40104_2016_100_MOESM3_ESM.pdf]

Table S3 Enriched GO terms (only significantly enriched GO terms were listed)

GO ID	Level	Description	Hits	Enrichmen	Genes	
GO:003024	3	carbohydrate binding	5	0.026	COLEC11, CXCL6, TINAG, SFTP	MF
GO:001545	2	auxiliary transport protein activity	2	0.046	SCN4B, DZIP3	MF
GO:001624	3	channel regulator activity	2	0.046	SCN4B, DZIP3	MF
GO:001085	3	cyclase regulator activity	1	0.045	GUCA1B	MF
GO:004442	2	extracellular region part	13	0.014	COLEC11, CXCL6, CCL19, BSP1,	CC
GO:000557	2	extracellular region	18	0.015	SPINK1, COLEC11, HTRA4, CXC	CC
GO: 005164	3	establishment of localization in cell	15	0.001	SCIN, RAB3A, MYLK2, GDAP1, I	BP
GO: 000695	3	immune response	11	0.002	CX3CR1, CXCL6, CCL19, BOLA-	BP
GO: 005164	3	cellular localization	15	0.003	SCIN, RAB3A, MYLK2, GDAP1, I	BP
GO: 000181	3	cytokine production	6	0.005	CXCL6, IL6, IL1B, IRF9, TLR7, IL	BP
GO: 000177	3	leukocyte homeostasis	3	0.007	CXCL6, IL6, IL2	BP
GO: 001972	3	cellular homeostasis	8	0.008	DZIP3, DHRS7C, IL6, SCN4B, TX	BP
GO: 003287	3	regulation of localization	10	0.01	SCIN, RAB3A, MYLK2, DHRS7C,	BP
GO: 005123	3	regulation of multicellular organismal pro	13	0.011	SCIN, RAB3A, MYLK2, CX3CR1,	BP
GO: 004233	3	taxis	4	0.011	CX3CR1, CXCL6, IL1B,CCL8	BP
GO: 000237	2	immune system process	13	0.012	SCIN, CX3CR1, CXCL6, CCL19, E	BP
GO: 005128	3	negative regulation of sequestering of calc	2	0.016	DHRS7C, DRD1	BP
GO: 003284	3	regulation of homeostatic process	3	0.025	DHRS7C, IL2, DRD1	BP
GO: 000165	3	temperature homeostasis	2	0.028	IL1B, DRD1	BP
GO: 005109	3	negative regulation of developmental proc	8	0.038	DZIP3, CX3CR1, KCNA3, IL1B, I	BP
GO: 000681	3	transport	25	0.04	SCIN, RAB3A, MYLK2, KCND3,	BP
GO: 005170	3	response to other organism	5	0.041	CXCL6, IL6, KRT8, IL1B, TLR7	BP
GO: 005123	3	maintenance of location	3	0.041	DHRS7C, IL1B, DRD1	BP
GO: 003250	2	multicellular organismal process	27	0.042	HOXC11, COLEC11, SCIN, GUCA	BP
GO: 005123	2	establishment of localization	25	0.043	SCIN, RAB3A, MYLK2, KCND3,	BP
GO: 002170	3	developmental maturation	3	0.044	RAB3A, BSP1, STXBP1	BP
GO: 006500	3	regulation of biological quality	15	0.047	DZIP3, SCIN, RAB3A, HTRA4, C	BP
