# Supplementary material for: High-concentrate feeding upregulates the expression of inflammation-related genes in the ruminal epithelium of dairy cattle
Source: J Anim Sci Biotechnol. 2016 Jul 29;7:42. doi: 10.1186/s40104-016-0100-1 (PMC4966727; doi:10.1186/s40104-016-0100-1)
Supplement: Additional file 4: Table S4. — DEGs were assigned to KEGG pathway. (PDF 29 kb) [file 40104_2016_100_MOESM4_ESM.pdf]

Table S4 DEGs were assigned to KEGG pathway

Pathway name	Enrichment test p value	q value	Molecules
Cytokine-cytokine receptor interaction	0	0	CCL19, CCL8, CX3CR1, CXCL6, IL15RA, IL1B, IL2, IL22, IL6, INHBE, LEPR, PRL, TNFRSF9
Graft-versus-host disease	4.00E-04	0.001	BOLA-DYA, IL1B, IL2, IL6
Jak-STAT signaling pathway	5.00E-04	0.001	IL15RA, IL2, IL22, IL6, IRF9, LEPR, PRL
Intestinal immune network for IgA produc	0.0011	0.002	BOLA-DYA, IL15RA, IL2, IL6
Type I diabetes mellitus	0.0076	0.009	BOLA-DYA, IL1B, IL2
Hematopoietic cell lineage	0.0081	0.009	CR2, IL1B, IL6, ITGB3
NOD-like receptor signaling pathway	0.0177	0.017	CCL8, IL1B, IL6
Calcium signaling pathway	0.0244	0.02	DRD1, HTR7, MYLK2, PLCB4, RYR3
Chemokine signaling pathway	0.0276	0.021	CCL19, CCL8, CX3CR1, CXCL6, PLCB4
Prion diseases	0.043	0.029	IL1B, IL6
Allograft rejection	0.0474	0.029	BOLA-DYA, IL2
Neuroactive ligand-receptor interaction	0.0676	0.037	ADCYAP1R1, DRD1, GRIA3, HTR7, LEPR, PRL
Toll-like receptor signaling pathway	0.0756	0.037	IL1B, IL6, TLR7
Autoimmune thyroid disease	0.0796	0.037	BOLA-DYA, IL2
Hedgehog signaling pathway	0.0823	0.037	IHH, WNT11
Axon guidance	0.0946	0.039	EFNB3, MGC139448, SEMA7A
Cytosolic DNA-sensing pathway	0.0991	0.039	IL1B, IL6
Regulation of actin cytoskeleton	0.117	0.043	FGF23, ITGB3, MYLK2, SCIN
Leishmaniasis	0.132	0.046	BOLA-DYA, IL1B
Long-term depression	0.1413	0.047	GRIA3, PLCB4
Hypertrophic cardiomyopathy (HCM)	0.1669	0.053	IL6, ITGB3
Gap junction	0.1898	0.058	DRD1, PLCB4
Asthma	0.2057	0.06	BOLA-DYA
Melanogenesis	0.2165	0.06	PLCB4, WNT11
Alzheimer's disease	0.2264	0.06	IL1B, PLCB4, RYR3
Cysteine and methionine metabolism	0.2689	0.065	SDS
Glycine, serine and threonine metabolism	0.2689	0.065	SDS
Taste transduction	0.2756	0.065	SCNN1B
Starch and sucrose metabolism	0.2889	0.065	TREH

SNARE interactions in vesicular transport	0.3019	0.065	VAMP1
Vascular smooth muscle contraction	0.3147	0.065	MYLK2, PLCB4
Aldosterone-regulated sodium reabsorptio	0.3209	0.065	SCNN1B
N-Glycan biosynthesis	0.3209	0.065	ST6GAL1
Retinol metabolism	0.3455	0.067	DHRS9
Basal cell carcinoma	0.3921	0.067	WNT11
Wnt signaling pathway	0.4068	0.067	PLCB4, WNT11
Inositol phosphate metabolism	0.4087	0.067	PLCB4
Amyotrophic lateral sclerosis (ALS)	0.4195	0.067	NEFH
MAPK signaling pathway	0.4461	0.067	FGF23, IL1B, PTPN5
Long-term potentiation	0.4508	0.067	PLCB4
Viral myocarditis	0.4558	0.067	BOLA-DYA
Arrhythmogenic right ventricular cardiom	0.4608	0.067	ITGB3
Melanoma	0.4608	0.067	FGF23
Adipocytokine signaling pathway	0.4658	0.067	LEPR
PPAR signaling pathway	0.4707	0.067	FABP2
B cell receptor signaling pathway	0.4804	0.067	CR2
Antigen processing and presentation	0.4899	0.067	BOLA-DYA
Complement and coagulation cascades	0.4899	0.067	CR2
ECM-receptor interaction	0.5084	0.067	ITGB3
Phosphatidylinositol signaling system	0.5084	0.067	PLCB4
Focal adhesion	0.5185	0.067	ITGB3, MYLK2
Dilated cardiomyopathy	0.5262	0.067	ITGB3
TGF-beta signaling pathway	0.5305	0.067	INHBE
Pathways in cancer	0.5575	0.068	FGF23, IL6, WNT11
Fc gamma R-mediated phagocytosis	0.5599	0.068	SCIN
Apoptosis	0.572	0.068	IL1B
GnRH signaling pathway	0.5875	0.069	PLCB4
T cell receptor signaling pathway	0.6274	0.072	IL2
Leukocyte transendothelial migration	0.6571	0.074	TXK
Cell adhesion molecules (CAMs)	0.6988	0.077	BOLA-DYA
Spliceosome	0.7043	0.077	THOC1

Purine metabolism	0.7588	0.082	AMPD1
Olfactory transduction	0.7787	0.082	GUCA1B, LOC526286, LOC540082, OR2D2
Huntington's disease	0.8304	0.086	PLCB4
Systemic lupus erythematosus	0.8425	0.086	BOLA-DYA
Metabolic pathways	0.9698	0.098	AMPD1, DHRS9, PLCB4, SDS, ST6GAL1
